# Effects of non-supervised low intensity aerobic excise training on the microvascular endothelial function of patients with type 1 diabetes: a non-pharmacological interventional study

**DOI:** 10.1186/s12872-016-0191-9

**Published:** 2016-01-27

**Authors:** Roger de Moraes, Diogo Van Bavel, Marília de Brito Gomes, Eduardo Tibiriçá

**Affiliations:** National Institute of Cardiology, Rio de Janeiro, Brazil; Laboratory of Cardiovascular Investigation, Oswaldo Cruz Institute, Av. Brasil, 4365, 21045-900 Rio de Janeiro, Brazil; Department of Medicine, Diabetes Unit, State University of Rio de Janeiro, Rio de Janeiro, Brazil; School of Physical Education and Sports Sciences of the Estácio de Sá University, Rio de Janeiro, Brazil

**Keywords:** Exercise training, Microvascular rarefaction, Endothelial dysfunction, Laser Doppler flowmetry

## Abstract

**Background:**

The aim of the present study was to evaluate changes in microvascular density and reactivity in patients with type 1 diabetes (T1D) resulting from low intensity chronic exercise training.

**Methods:**

This study included 22 (34 ± 7 years) consecutive outpatients with T1D and disease duration > 6 years. We used intravital video-microscopy to measure basal skin capillary density and capillary recruitment using post-occlusive reactive hyperemia (PORH) in the dorsum of the fingers. Endothelium-dependent and -independent vasodilation of the skin microcirculation was evaluated in the forearm with a laser Doppler flow monitoring (LDF) system in combination with acetylcholine and sodium nitroprusside iontophoresis, PORH and local thermal hyperemia.

**Results:**

The basal mean capillary density (MCD) after exercise training was significantly higher than before exercise (134 ± 25 vs. 119 ± 19 capillaries/mm^2^, respectively; *P* = 0.0013). MCD during PORH was also higher after exercise (140 ± 26 vs. 121 ± 24 capillaries/mm^2^, respectively; *P* < 0.0001). Endothelium-dependent capillary recruitment during PORH was also significantly higher after exercise (140 ± 26 vs. 134 ± 25 capillaries/mm^2^, respectively; *P* < 0.0012). There were no significant changes in skin microvascular reactivity after exercise as investigated using LDF.

**Conclusions:**

Our results showed that low intensity aerobic exercise, performed four times per week for 12 weeks by patients with T1D, induces significant increases in microvascular density and endothelial-dependent capillary reactivity.

**Trial registration:**

ClinicalTrials.gov NCT02441504. Registered 7 May 2015.

**Electronic supplementary material:**

The online version of this article (doi:10.1186/s12872-016-0191-9) contains supplementary material, which is available to authorized users.

## Background

Type 1 diabetes mellitus (T1D) is an autoimmune disease whose clinical manifestations are characterized by extensive damage to the macro and microcirculation. It is believed that the loss of glycemic control that occurs during the disease is responsible for functional and structural vascular alterations, contributing to significant increases in the risk of morbidity and mortality in this group of individuals [1–4].

Through the induction of oxidative stress, inflammation and the formation of nonenzymatic advanced glycation end products (AGEs), hyperglycemia can cause microvascular abnormalities that impair tissue nutrition [5, 6]. In this context, from the beginning of the disease, patients with T1D present with widespread precapillary vasodilation and tissue hypoxia, which promotes compensatory increases in the level of vasoactive substances and chronic elevation of the microvascular blood flow, resulting in morphological and functional changes associated with capillary rarefaction [7–12].

In spite of the positive effects of anti-hyperglycemic therapy [13] and drugs that attenuate inflammation and oxidative stress [14], chronic aerobic exercise has been shown to exert anti-inflammatory, anti-hyperglycemic and anti-oxidant effects [15]. Moreover, exercise training contributes to an increase in capillary density in patients with T1D [16]. In fact, aerobic training is often incorporated into T1D treatment therapies [17–20] because it can prevent undesirable morphological changes and, through the processes of arteriogenesis and angiogenesis, restore and expand the vascular reactivity and tissue perfusion that are otherwise impaired by the disease [21–24].

Several studies have demonstrated that aerobic training with intensity exceeding 50 % of VO_2max_ can improve macro- and microvascular endothelial function in young people with T1D [25–27], suggesting that the microvascular function in patients with T1D is directly proportional to the individual level of aerobic fitness [28]. However, it is still not clear whether the effects of low intensity exercise intervention, which is considered to be safer than higher intensity intervention, are more suited to the average functional capacity of this population. Considering the therapeutic potential of aerobic training to promote metabolic and vascular adaptations that prevent the development of clinical complications in T1D patients, this study aimed to investigate the effects of 12 weeks of low intensity aerobic exercise on microvascular reactivity in this group of individuals.

## Methods

### Subjects

The present study was performed in accordance with the Helsinki Declaration of 1975, as revised in 2000, and the Institutional Review Board (IRB) of the University Hospital of the State University of Rio de Janeiro, Brazil approved of this study. Once considered eligible, all subjects read and signed an informed consent document that was approved by the IRB.

Study subjects presenting with type 1 diabetes (diagnosis based on a typical clinical presentation as well as the need to use insulin continuously since the diagnosis) of both sexes were recruited among patients who were followed up at a public hospital. Twenty-eight individuals with T1D diagnosed for more than 6 years, between 25 and 50 years of age, were recruited for the study. Twenty-two individuals (mean age 34 ± 7 years) completed the exercise protocol. There were 13 males (60 %) and 9 females (40 %) in the study group. The other six subjects (21 % of the subjects initially recruited for the study) left the study due to personal reasons.

The initial clinical evaluation included the patient’s history and physical examination as well as recording of the following anthropometric data: weight, height, waist circumference and body mass index (BMI). Blood pressure measurements were collected with patients in the supine position after 5 min in quiet surroundings; they were repeated twice with a two-minute interval between measurements. All measurements were performed before and after 12 weeks of physical training.

All patients included in the study were advised to maintain the same level of usual daily physical activity during the experimental protocol. Moreover, a regular diet for patients with type 1 diabetes was prescribed and supervised by the clinical staff of the tertiary care setting where they were recruited to certify that no significant changes occurred in the composition or total calories of the diet.

The diabetic patients included in the study were relatively young and did not present any co-morbidity. They were treated exclusively with insulin for diabetes control by an endocrinologist in the tertiary care setting, and none of them was taking other medications.

### Blood sampling and laboratory tests

On the morning scheduled for the evaluation of cutaneous microcirculation, the patients presented in 12-h fasted condition for blood collection. Smokers should not have smoked or ingested caffeine from the night before until the completion of the tests. The following variables were measured: fasting and postprandial plasma glucose, total cholesterol, LDL and HDL cholesterol, triglycerides, transaminases, high-sensitivity C-reactive protein, gamma-glutamyl transferase, creatine kinase, urea, creatinine, albumin and uric acid by colorimetric reactions by using a Cobas Mira-machine (Roche). Blood samples were collected before and after the physical training period. LDL cholesterol was calculated using Friedewald’s formula. Serum samples were kept frozen at –80 °C until measurement of interleukin-6 (IL-6) levels with a commercial ELISA kit (Cayman Chemical Company, Ann Arbor, MI, USA) according to the manufacturer’s instructions.

### Physical training

The study participants followed a non-supervised aerobic training protocol targeted at low intensity and corresponding to 40 % of their heart rate (HR) reserve (HRR). Resting HR was evaluated at the time of assessment of microvascular reactivity, and maximum HR was calculated using the formula [208 – (0.7 × age)] as described by Tanaka and colleagues [29]. HRR was calculated as proposed by Karvonen [30] [(HRmax – HRrest) x 40 % + HRrest] because it is highly correlated with VO_2_ maximum [31]. The patients were advised to perform exercise sessions four times per week for 12 weeks that included alternating walking and running, in accordance with the patient’s fitness level, corresponding to 40 % of the HRR. Follow-up of the training period was conducted through weekly telephone contact. Heart rate was monitored using heart rate monitors (Polar Electro Oy, Kempele, Finland). During the first 4 weeks of training, 10 min were added per session every week to promote gradual progression in the range from 30 to 60 min during the remaining 8 weeks.

### Capillaroscopy by intra-vital videomicroscopy

Microcirculatory tests were always performed after a 20-min period of rest in the supine position in a temperature-controlled room (23 ± 1 °C), thus excluding the influence of climate conditions from the results of the tests.

The capillary density, defined as the number of perfused capillaries per mm^2^ of skin area, was assessed by high-resolution intra-vital color microscopy (Moritex, Cambridge, UK) using a video microscopy system with an epi-illuminated fiber optic microscope containing a 100-W mercury vapor lamp light source and an M200 objective with a final magnification of 200×. The dorsum of the non-dominant middle phalanx was used for image acquisition, which occurred while the patient sat comfortably in a constant temperature environment (23 ± 1 °C), as described in detail by Antonios et al. [32–34]. Images were acquired and saved for posterior off-line analysis using a semi-automatic integrated system (Microvision Instruments, Evry, France). The mean capillary density was calculated as the arithmetic mean of the number of visible (i.e., spontaneously perfused) capillaries in three contiguous microscopic fields of 1 mm^2^ each. A blood pressure cuff was then applied to the patient’s arm and inflated to suprasystolic pressure (50 mmHg above systolic arterial pressure) to completely interrupt blood flow for three minutes (testing post-occlusive reactive hyperemia, PORH). After cuff release, images were again acquired and recorded during the following 60–90 s, during which a maximal hyperemic response was expected to occur.

We assessed the variability of skin video capillaroscopy in a previous study [12]. The reproducibility of this methodology was assessed by examining an identical area of the finger skin; the intra-observer repeatability of data analysis was assessed by reading the same images in a blinded manner on two separate occasions (*n* = 20; coefficient of variability, 4.3 %). To assess inter-observer repeatability, a second observer independently assessed the capillary density in the same images (*n* = 15; coefficient of variability, 3.3 %).

### Microvascular reactivity to pharmacological stimulation

Microvascular cutaneous reactivity was studied by laser Doppler flow monitoring (LDF), a method that has previously been standardized and validated [10, 35, 36]. Real-time variations in cutaneous microcirculatory flow were assessed through an LDF system (wavelength 780 nm; Periflux 5001, Perimed AB, Järfälla, Sweden) attached to a pharmacological micro-iontophoresis system (PeriIont, Perimed AB). The iontophoresis microelectrodes (PF 383, Perimed) were incorporated into the head of a laser probe (PF 481–1, Perimed), and the probe temperature was standardized to 32^°^C to avoid variations in skin temperature and, consequently, in the measurements of microvascular flow. The drug-delivery electrodes were filled with 200 μl of 1 % ACh solution (Sigma Chemical Co., USA) and placed on the ventral surface of the forearm, away from visible subcutaneous veins and areas of skin pigmentation. Neutral electrodes for current dispersion were placed 15 cm above the infusion electrodes, and reference points were marked and annotated to ensure reproducibility during the second examination, which took place at the end of the intervention period. After measuring the baseline flow for 5 min, four equal cumulative doses of ACh (anodal current) were applied at a constant intensity of 0.1 mA for 10, 20, 40 and 80 s, with total charges of 1, 2, 4 and 8 millicoulombs, respectively, allowing for a 120-s interval between doses. Recording of the microvascular flow induced by ACh was conducted for 10 min as follows: 2 min for each dose and 2 min to allow the flow to reach a plateau after the last dose. Using a new delivery electrode applied to a different location on the same forearm, four doses of a solution of 1 % sodium nitroprusside (SNP; Sigma Chemical CO, USA) dissolved in distilled water were delivered using a cathodal current (same charges and intervals as for ACh).

The laser Doppler output, which is semiquantitative, is expressed in arbitrary perfusion units (PUs) of output voltage (1 PU = 10 mV) in accordance with general consensus (European Laser Doppler Users Groups, London, 1992). An area under the flow response to ACh curve could be defined using the PeriSoft for Windows 2.5 software (Perimed, Järfälla, Sweden); this area was quantitatively measured and expressed in PU/s.

### Microvascular reactivity to physiologic stimulation

After measuring the resting capillary flow for 5 min using another laser probe (PF 457, Perimed) that had been positioned at the start of the recording session, the PORH test was performed on the same forearm of pharmacological stimulation. Following release of the pressure, the maximum flow and area under the PORH curve were measured. The mean value of the resting flow was considered the basal flow value. When the microvascular flow returned to its basal value after the PORH test (typically 5–10 min), the maximal skin microvascular vasodilatation was investigated with prolonged (20 min) local heating of the laser probe to 42^°^C. The baseline microvascular flux value was calculated as described above.

### Statistical analysis

The values were expressed as the means ± standard error of the means for variables with a normal distribution and as median (percentiles 25^th^–75^th^) for variables with a non-parametric distribution according to results of the Shapiro-Wilk normality test. The data were analyzed by two-tailed paired *t* tests or the two-tailed Wilcoxon signed-rank test as appropriate. The null hypothesis was rejected at *P* < 0.05.

## Results

The clinical and laboratory variables of the patients at baseline and post-exercise training are presented in Table 1. We observed significant reductions in body weight and body mass index, as well as in uric acid and interleukin-6 serum levels, after 12 weeks of exercise training.

**Table 1:** Clinical characteristics of the patients with type 1 diabetes before and after exercise training

Characteristics	Before training	After training	*P* value
	(n = 22)	(n = 22)	
Body weight (kg)	68.5 ± 15.3	67.7 ± 14.7*	**0.0247**
Body mass index (kg/m^2^)	24.2 ± 4.3	23.9 ± 4.1*	**0.0259**
Systolic arterial pressure (mmHg)	124.1 ± 19.7	123.2 ± 18.5	0.6753
Diastolic arterial pressure (mmHg)	79.3 ± 12.3	76.1 ± 14.0	0.0641
Mean arterial pressure (mmHg)	93.7 ± 14.8	92.9 ± 14.7	0.6004
Heart rate (bpm)	74.1 ± 11.1	77.6 ± 12.2	0.1398
Creatinine (mg/dl)	0.88 ± 0.17	0.82 ± 0.19	0.1671
Urea (mg/dl)	31.5 ± 9.6	33.4 ± 11.2	0.3362
Uric acid (mg/dl)	4.4 ± 1.2	3.8 ± 1.5*	**0.0198**
Fasting plasma glucose (mg/dl)	171.7 ± 75.2	169.0 ± 93.7	0.9076
Postprandial plasma glucose (mg/dl)	217.9 ± 79.5	221.5 ± 103.5	0.8940
HbA1c (%)	8.6 ± 1.7	9.0 ± 2.4	0.3278
Total cholesterol (mg/dl)	176.6 ± 30.2	191.1 ± 53.7	0.0966
HDL-C (mg/dl)	55.2 ± 16.5	57.3 ± 20.7	0.4217
LDL-C (mg/dl)	99.5 ± 6.1	111.1 ± 10.1	0.1762
Triglycerides (mg/dl)	109.5 ± 72.5	113.3 ± 82.9	0.8234
Albumin (g/dl)	4.2 ± 0.42	4.1 ± 0.5	0.2639
AST (U/L)	23.3 ± 9.9	20.3 ± 6.1	0.1966
ALT (U/L)	19.7 ± 7.6	19.9 ± 10.7	0.9122
GGT (IU/L)	126.7 ± 51.6	147.5 ± 81.6	0.1867
CK-MM (U/L)	20.25 ± 2.0	19.4 ± 3.1	0.7992
hs-CRP (mg/dL)	0.37 ± 0.76	0.29 ± 0.32	0.5685
Interleukin-6 (ng/ml)	8.5 ± 3.1	4.5 ± 2.6*	**0.0052**

The results are presented as the means ± SEM

*P* values were estimated using two-tailed paired Student’s *t* tests; bold values represent statistically significant differences

*P < 0.05 vs. values before training

ALT: alanine transaminase; AST: aspartate transaminase; CK-MM: creatine kinase-MM; GGT, gamma-glutamyl transferase; HbA1c, glycated hemoglobin; hs-CRP: high-sensitivity C-reactive protein; HDL-C: high-density lipoprotein cholesterol; and LDL-C: low-density lipoprotein cholesterol

### Capillary density

**Fig. 1:**
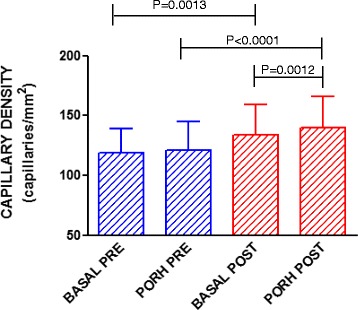
Functional capillary density at baseline (BASAL) and during post-occlusive reactive hyperemia (PORH) before (PRE) and after (POST) exercise training in patients with type 1 diabetes (*n* = 22). The values represent the means ± SEM. Paired or unpaired Student’s two-tailed *t*-tests were used when appropriate

The mean capillary density after exercise training was significantly higher than before training (Fig. 1). A similar pattern was found during the PORH test; the capillary density was significantly higher after the training period compared with before the training period (Fig. 1). The increase in the capillary density measured in the basal state was from 119 ± 19 to 134 ± 25 capillaries/mm^2^ (*P* = 0.0013) and after PORH from 121 ± 24 to 140 ± 26 capillaries/mm^2^ (*P* < 0.0001). Moreover, there was improvement in the capillary recruitment induced by the PORH test after training; the capillary density after the PORH test increased from 134 ± 25 to 140 ± 26 capillaries/mm^2^ (*P* = 0.0012).

### Microvascular reactivity to pharmacological and physiological stimuli

**Fig. 2:**
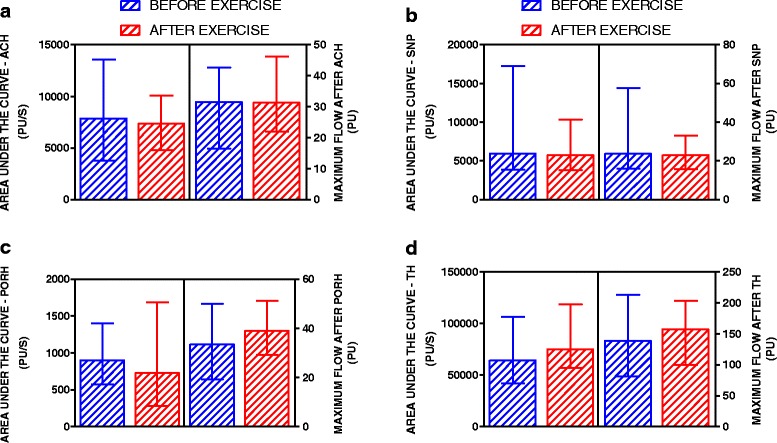
Microcirculatory parameters of patients with type 1 diabetes before and after exercise training. The maximum microvascular blood flow, expressed in arbitrary perfusion units (PU), and the area under the curve, expressed in PU/s, resulted from microvascular stimulation with acetylcholine (**a**, ACH), sodium nitroprusside (**b**, SNP), post-occlusive reactive hyperemia (**c**, PORH) and thermal hyperemia (**d**, TH)

The skin microvascular vasodilation responses induced by either endothelial-dependent (ACh) or endothelial-independent (SNP) vasoactive drugs were not different before and after exercise training (Fig. 2). The same pattern of responses was observed after physiological endothelial-dependent stimulation with the PORH test and local heating (Fig. 2). The individual values of microvascular parameters obtained with LDF are presented in Additional file 1.

## Discussion

The results of the present study indicate that 3 months of non-supervised aerobic exercise training performed at 40 % of HRR significantly increased the microvascular density of T1D patients and reduced their BMI and uric acid serum levels. The low intensity aerobic training program also improved basal capillary density and capillary recruitment during post-occlusive reactive hyperemia, suggesting that improvements occurred in systemic capillary number and perfusion.

It is well demonstrated that elevated uric acid serum levels are associated with endothelial dysfunction and renal microvascular complications in patients with T1D [36–39]. Although the patients’ serum uric acid levels were within the normal range, low-intensity aerobic training was able to promote a significant reduction that could be associated with increased capillary density. Such changes may have contributed to expanding the supply of tissue nutrients and reducing the catabolism of adenine as well as the rate of uric acid formation [40, 41].

Despite previous contradictory reports [42, 43], our results showed that aerobic activity of low difficulty level, performed within the functional capacity of sedentary individuals, can promote significant increases in the microvascular density of T1D patients, who appear to have a severely compromised angiogenic process in the absence of exercise [44, 45]. In fact, as shown by previous studies [12, 46], and considering that sedentary T1D patients do not present with a capillary reserve in either the hands or feet [12], the changes in capillary density observed in the present study after exercise training suggest that low intensity aerobic exercise not only represents an important stimulus for increased capillary perfusion, it also contributes to increasing the number of capillaries.

In our study, the improvement in capillary perfusion and function was not accompanied by significant changes in microvascular reactivity after exercise training. It is conceivable that the low intensity exercise training did not induce sufficient increases in blood flow velocity and vascular shear stress required to significantly increase the release and bioavailability of vasoactive molecules, such as nitric oxide. In fact, higher intensity physical exercise has already been shown to induce significant increases in cutaneous microvascular reactivity [47, 48]. On the other hand, vascular growth and remodeling and enhanced angiogenesis, i.e., capillary growth, are well-known features of physiological adaptations to chronic exercise and have been extensively reviewed [24, 49].

Low-to-moderate intensity aerobic exercise has been associated with lower oxidative damage to the vascular endothelium [50–52] as well as with selective increases in angiogenic factors such as vascular endothelial growth factor (VEGF) and hypoxia-inducible factor (HIF-1α) [42, 53]. On the other hand, it is possible that the average intensity of 40 % of the heart rate reserve used in our study corresponds to an increase in the ventricular ejection fraction that is not associated with high sympathetic activation, which could have beneficial effects on capillary perfusion without imposing harmful interference associated with increases in arteriolar tone. Therefore, it is reasonable to speculate that during each workout, there was an increase in the supply of humoral factors, such as atrial natriuretic peptide, that are capable of stimulating perfusion and/or increasing capillary density [54]. We speculate this in spite of the absence of a training effect on endothelium-dependent or -independent microvascular reactivity, as measured by laser Doppler flowmetry, which is most likely caused by insufficient increases in the flow velocity and vascular shear stress needed to increase the bioavailability of vasoactive molecules such as nitric oxide [55]. The absence of significant differences in microvascular reactivity, as measured by single point laser Doppler methodology, could be related to the high variability of the vasodilator effects obtained upon cutaneous iontophoresis with acetylcholine and sodium nitroprusside.

In this context, there is evidence that an increase in plasma free fatty acid (FFA) levels, which predominantly occurs with exercise intensity below 50 % of VO_2max_ [56–58], can activate peroxisome proliferator-activated receptor beta or delta (PPAR-β or PPAR-δ), as well as PPAR-δ coactivator-alpha (PGC-1α), contributing to the expression of VEGF and consequent development of the angiogenic process [59–61]. Nevertheless, we do not have data about plasma levels of VEGF or of its soluble receptor FLT-1 in the patients with T1D, and thus we cannot conclude that this mechanism is involved in the increases in systemic capillary observed after exercise training.

On the other hand, it has been suggested in animal studies that low intensity exercise can activate PGC-1α [62, 63] independently of 5′ adenosine monophosphate-activated protein kinase (AMPK) activation, which might only occur with exercise of higher intensity [64].

Increases in body temperature have been associated with improvements in tissue perfusion and capillary angiogenesis in animals and humans [65, 66]. In our study, the average temperature of the T1D patients during exercise was 37.8 ± 0.8^°^C, which may have been an important stimulus for the changes in capillary perfusion. In this sense, because it has been shown that physiological levels of urate are needed to promote increases in capillary perfusion in thermal stress situations [67], it is reasonable to speculate that the decrease in uric acid levels represents improvements in perfusion associated with the adaptive effects of low intensity training and resulting in capillary angiogenic processes.

Our results also indicated that low intensity aerobic training was able to restore the ability of capillary self-regulation at rest. In fact, studies with diabetic animals had already suggested that, through stimulating angiogenesis, aerobic training was able to partially restore the capillary diffusion capacity and regional distribution reduced by hyperglycemia [44] and losses in capillary perfusion [44, 68, 69].

Training methodologies that combine low intensity aerobic exercise in situations that mimic the reduction of tissue nutrients present in patients with T1D, such as venous occlusion training techniques, have been shown to result in significant metabolic adaptations associated with improvements in insulin sensitivity and muscle strength [70]. Moreover, despite the proposition that high intensity interval training can promote increases in the capillary density of skeletal muscle, this exercise intensity has recently been suggested to be small compared to lower exercise intensities [71, 72].

It is well established that interplay between inflammatory and metabolic alterations leads to vascular injury in diabetes, which is mainly represented by microvascular endothelial dysfunction [73–75]. Therefore, an array of circulating biomarkers of systemic subclinical chronic inflammation has been investigated to target therapy in diabetes. Interleukin 6 (IL-6) appears to be a systemic inflammatory marker that is correlated with the degree of inflammatory activity in both T1D and T2D [74, 76]. In our study, plasma IL-6 levels were markedly and significantly reduced after exercise, indicating that low intensity aerobic exercise is an efficient non-pharmacologic intervention affecting the vascular inflammatory profile of T1D patients. This effect could also explain the improvement in endothelium-dependent capillary recruitment observed after exercise training.

It is important to note that in addition to its well-known pro-inflammatory actions, IL-6 can also have an anti-inflammatory role. Multiple cytokines, including IL-6, IL-1, and TNF-α, are consistently elevated in inflammatory states and have been recognized as targets of therapeutic intervention. Nevertheless, IL-6 can also play an anti-inflammatory role in both local and systemic inflammatory responses by controlling levels of the pro-inflammatory cytokines TNF-α and IL-1 [77]. In our study, the reductions in IL-6 plasma levels observed after exercise training suggests that this non-pharmacologic intervention is able to reduce the low-grade systemic inflammation that is typical of T1D patients. In future studies, comprehensive evaluations of plasma levels of different pro- and anti-inflammatory cytokines are warranted to investigate the putative interactions between these different cytokines.

### Limitations and strengths of the study

The present study has limitations that may have influenced the findings. Such limitations include the fact that there was no control (sedentary) group of patients. Considering that physical exercise is a classical non-pharmacologic intervention in cardiovascular and metabolic diseases, we consider that it could be unethical to have a group of patients not participating in the exercise training program. On the other hand, we could have used a control (run in) period when the patients could have been evaluated after a period of inactivity. However, the main drawback of this type of experimental design is the resulting decrease of adherence to the study protocol, which is typical of patients with chronic diseases, and could have resulted in a greater dropout rate from the study.

Finally, it is also worth mentioning that the increases in systemic capillarity and improvement of capillary recruitment in patients with type 1 diabetes were obtained with an exercise training protocol that can be easily performed in the everyday life of patients with chronic diseases.

## Conclusions

Our results showed that non-supervised low intensity aerobic exercise, performed four times per week for 12 weeks by patients with type 1 diabetes, induces significant increases in microvascular density and endothelial-dependent capillary reactivity. Microvascular improvement in patients with diabetes is essential for preventing complications and targeting end-organ damage.

## Additional file

Additional file 1:
**Supplementary data tables.** (ZIP 671 kb)

## Abbreviations

ALT: Alanine transaminase
AST: Aspartate transaminase
AGEs: Advanced glycation end products
BMI: Body mass index
CK-MM: Creatine kinase-MM
GGT: Gamma-glutamyl transferase
HbA1c: Glycated hemoglobin
HDL-C: High-density lipoprotein cholesterol
hs-CRP: High-sensitivity C-reactive protein
LDL-C: Low-density lipoprotein cholesterol
LDF: Laser Doppler flow monitoring
MCD: Mean capillary density, PORH, Post-occlusive reactive hyperemia
T1D: Type 1 diabetes
