# Supplementary material for: Effects of non-supervised low intensity aerobic excise training on the microvascular endothelial function of patients with type 1 diabetes: a non-pharmacological interventional study
Source: BMC Cardiovasc Disord. 2016 Jan 27;16:23. doi: 10.1186/s12872-016-0191-9 (PMC4728937; doi:10.1186/s12872-016-0191-9)
Supplement: Additional file 1: — Supplementary data tables. (ZIP 671 kb) [file 12872_2016_191_MOESM1_ESM.zip › 4578932131633087_add1.pdf]

Supplementary data table 1: Individual values for microcirculatory parameters of the patients with type 1 diabetes before and after exercise training. The peak values of microvascular flow resulting from acetylcholine administration are expressed in arbitrary perfusion units.

	**ACETYLCHOLINE-MEDIATED PEAK INCREASES IN FLOW (perfusion units)**	
**Study subject**	**BEFORE EXERCISE**	**AFTER EXERCISE**
1	79.38	44.68
2	58.84	75.47
3	15.26	35.06
4	19.93	19.07
5	33.05	49.16
6	13.03	41.08
7	34.21	27.44
8	39.55	76.32
9	14.01	26.12
10	16.75	16.76
11	31.73	25.48
12	38.33	22.98
13	7.02	16.75
14	58.38	45.26
15	32.01	29.63
16	25.51	51.85
17	28.41	40.37
18	65.40	117.49
19	25.36	10.41
20	31.34	32.87
21	13.46	8.88
22	51.91	24.81
