# Supplementary material for: Effects of non-supervised low intensity aerobic excise training on the microvascular endothelial function of patients with type 1 diabetes: a non-pharmacological interventional study
Source: BMC Cardiovasc Disord. 2016 Jan 27;16:23. doi: 10.1186/s12872-016-0191-9 (PMC4728937; doi:10.1186/s12872-016-0191-9)
Supplement: Additional file 1: — Supplementary data tables. (ZIP 671 kb) [file 12872_2016_191_MOESM1_ESM.zip › 4578932131633087_add2.pdf]

Supplementary data table 2: Individual values for microcirculatory parameters of the patients with type 1 diabetes before and after exercise training. The area under the curve of microvascular flow increases resulting from acetylcholine administration is expressed in perfusion units/s.

	AREA UNDER THE CURVE OF ACETYLCHOLINE (perfusion units/s)	
**Study subject**	**BEFORE EXERCISE**	**AFTER EXERCISE**
1	14,170.40	10,457.37
2	9,676.54	22,001.15
3	3,621.61	7,442.37
4	3,411.09	6.934.69
5	8,957.86	10,049.13
6	3,815.63	5,701.30
7	6,754.66	4,410.92
8	14,058.81	10,025.06
9	3,558.15	8,816.91
10	2,976.29	4,782.76
11	6,474.42	4,036.55
12	13,424.16	7,726.37
13	3,503.68	4,807.61
14	14,132.84	9,805.57
15	9,250.86	5,658.58
16	5,049.03	16,144.10
17	5,468.15	6,005.42
18	23,287.01	41,837.38
19	9,376.20	2,257.71
20	12,192.96	10,097.28
21	5,124.18	2,218.07
22	14,628.27	7,206.96
