# Supplementary material for: Effects of non-supervised low intensity aerobic excise training on the microvascular endothelial function of patients with type 1 diabetes: a non-pharmacological interventional study
Source: BMC Cardiovasc Disord. 2016 Jan 27;16:23. doi: 10.1186/s12872-016-0191-9 (PMC4728937; doi:10.1186/s12872-016-0191-9)
Supplement: Additional file 1: — Supplementary data tables. (ZIP 671 kb) [file 12872_2016_191_MOESM1_ESM.zip › 4578932131633087_add3.pdf]

Supplementary data table 3: Individual values for microcirculatory parameters of the patients with type 1 diabetes before and after exercise training. The peak values of microvascular flow resulting from sodium nitroprusside administration are expressed in arbitrary perfusion units.

	**SODIUM NITROPRUSSIDE - MEDIATED PEAK INCREASES IN FLOW (perfusion units)**	
**Study subject**	**BEFORE EXERCISE**	**AFTER EXERCISE**
1	62.23	35.16
2	46.88	92.71
3	16.02	56.21
4	19.56	26.86
5	65.52	68.54
6	15.35	27.34
7	34.21	22.31
8	25.12	22.77
9	22.16	9.77
10	5.43	23.10
11	64.45	21.91
12	64.82	5.37
13	19.41	17.12
14	13.12	29.97
15	19.29	78.77
16	83.83	32.44
17	37.20	15.90
18	56.15	15.20
19	4.15	5.86
20	16.75	23.01
21	11.63	17.15
22	47.85	15.11
