# Supplementary material for: Effects of non-supervised low intensity aerobic excise training on the microvascular endothelial function of patients with type 1 diabetes: a non-pharmacological interventional study
Source: BMC Cardiovasc Disord. 2016 Jan 27;16:23. doi: 10.1186/s12872-016-0191-9 (PMC4728937; doi:10.1186/s12872-016-0191-9)
Supplement: Additional file 1: — Supplementary data tables. (ZIP 671 kb) [file 12872_2016_191_MOESM1_ESM.zip › 4578932131633087_add4.pdf]

Supplementary data table 4: Individual values for microcirculatory parameters of the patients with type 1 diabetes before and after exercise training. The area under the curve of microvascular flow increases resulting from sodium nitroprusside administration is expressed in perfusion units/s.

	AREA UNDER THE CURVE OF SODIUM NITROPRUSSIDE (perfusion units/s)	
**Study subject**	**BEFORE EXERCISE**	**AFTER EXERCISE**
1	17,093.38	5,608.99
2	11,934.52	18,800.07
3	3,651.81	23,311.53
4	4,920.35	7,389.98
5	29,778.45	14,899.21
6	4,542.05	5,863.72
7	12,049.27	7,745.49
8	6,092.59	5,522.94
9	8,332.08	3,013.70
10	2,933.07	5,354.68
11	24,763.32	108,240.62
12	24,982.97	1,640.59
13	5,781.98	5,165.96
14	5,208.42	8,806.57
15	4,980.94	16,383.22
16	17,726.02	7,832.82
17	11,437.07	5,905.77
18	18,932.84	3,796.41
19	1,954.49	2,279.18
20	3,040.51	2,483.21
21	3,331.84	3,909.23
22	3,953.69	3,799.98
