# Supplementary material for: Effects of non-supervised low intensity aerobic excise training on the microvascular endothelial function of patients with type 1 diabetes: a non-pharmacological interventional study
Source: BMC Cardiovasc Disord. 2016 Jan 27;16:23. doi: 10.1186/s12872-016-0191-9 (PMC4728937; doi:10.1186/s12872-016-0191-9)
Supplement: Additional file 1: — Supplementary data tables. (ZIP 671 kb) [file 12872_2016_191_MOESM1_ESM.zip › 4578932131633087_add5.pdf]

Supplementary data table 5: Individual values for microcirculatory parameters of the patients with type 1 diabetes before and after exercise training. The peak values of microvascular flow resulting from post-occlusive reactive hyperemia (PORH) are expressed in arbitrary perfusion units.

	**PORH -MEDIATED PEAK INCREASES IN FLOW (perfusion units)**	
**Study subject**	**BEFORE EXERCISE**	**AFTER EXERCISE**
1	56.95	39.07
2	17.94	61.08
3	42.04	38.81
4	16.33	46.94
5	40.84	56.69
6	48.55	49.50
7	32.92	67.69
8	46.30	29.75
9	15.52	29.99
10	13.26	25.85
11	23.47	26.98
12	36.99	38.76
13	55.23	43.07
14	54.23	18.92
15	33.88	27.15
16	24.75	40.24
17	19.59	33.23
18	17.29	112.26
19	82.54	36.05
20	31.98	15.09
21	23.06	122.50
22	78.03	42.49
