# Supplementary material for: Effects of non-supervised low intensity aerobic excise training on the microvascular endothelial function of patients with type 1 diabetes: a non-pharmacological interventional study
Source: BMC Cardiovasc Disord. 2016 Jan 27;16:23. doi: 10.1186/s12872-016-0191-9 (PMC4728937; doi:10.1186/s12872-016-0191-9)
Supplement: Additional file 1: — Supplementary data tables. (ZIP 671 kb) [file 12872_2016_191_MOESM1_ESM.zip › 4578932131633087_add6.pdf]

Supplementary data table 6: Individual values for microcirculatory parameters of the patients with type 1 diabetes before and after exercise training. The area under the curve of microvascular flow increases resulting from post-occlusive reactive hyperemia (PORH) is expressed in arbitrary perfusion units/s.

	AREA UNDER THE CURVE OF PORH (perfusion units/s)	
**Study subject**	**BEFORE EXERCISE**	**AFTER EXERCISE**
1	1,498.27	713.08
2	626.29	1,928.02
3	941.09	1,159.09
4	1,381.85	1,475.54
5	1,815.56	1,734.66
6	1,152.26	1,928.29
7	4,467.62	4,009.66
8	881.58	412.04
9	307.02	449.63
10	211.10	248.70
11	589.59	283.65
12	1,479.37	135.76
13	917.46	334.10
14	1,457.19	273.11
15	1,159.17	742.98
16	1,017.93	1,670.15
17	574.88	1,128.58
18	749.32	840.25
19	569.30	234.34
20	104.52	164.43
21	105.81	3,905.45
22	692.33	510.44
