# Supplementary material for: Effects of non-supervised low intensity aerobic excise training on the microvascular endothelial function of patients with type 1 diabetes: a non-pharmacological interventional study
Source: BMC Cardiovasc Disord. 2016 Jan 27;16:23. doi: 10.1186/s12872-016-0191-9 (PMC4728937; doi:10.1186/s12872-016-0191-9)
Supplement: Additional file 1: — Supplementary data tables. (ZIP 671 kb) [file 12872_2016_191_MOESM1_ESM.zip › 4578932131633087_add7.pdf]

Supplementary data table 7: Individual values for microcirculatory parameters of the patients with type 1 diabetes before and after exercise training. The peak values of microvascular flow resulting from thermal hyperemia are expressed in arbitrary perfusion units.

	THERMAL HYPEREMIA-MEDIATED PEAK INCREASES IN FLOW (perfusion units)	
**Study subject**	**BEFORE EXERCISE**	**AFTER EXERCISE**
1	186.22	132.87
2	82.34	184.17
3	135.44	98.02
4	109.86	264.53
5	135.01	262.45
6	142.49	147.86
7	91.06	117.25
8	213.23	134.46
9	53.44	99.09
10	65.55	167.57
11	95.55	118.65
12	79.13	96.92
13	146.67	181.67
14	222.93	100.13
15	45.23	87.65
16	184.78	168.27
17	49.01	99.89
18	213.65	394.32
19	377.23	201.23
20	178.22	180.88
21	218.38	468.26
22	273.16	2086.40
