# Supplementary material for: Effects of non-supervised low intensity aerobic excise training on the microvascular endothelial function of patients with type 1 diabetes: a non-pharmacological interventional study
Source: BMC Cardiovasc Disord. 2016 Jan 27;16:23. doi: 10.1186/s12872-016-0191-9 (PMC4728937; doi:10.1186/s12872-016-0191-9)
Supplement: Additional file 1: — Supplementary data tables. (ZIP 671 kb) [file 12872_2016_191_MOESM1_ESM.zip › 4578932131633087_add8.pdf]

Supplementary data table 8: Individual values for microcirculatory parameters of the patients with type 1 diabetes before and after exercise training. The area under the curve of microvascular flow increases resulting from thermal hyperemia (TH) is expressed in perfusion units/s.

Study subject	AREA UNDER THE CURVE OF TH (perfusion units/s) BEFORE EXERCISE	AFTER EXERCISE
1	112,869.70	75,042.08
2	55,480.80	114,525.30
3	81,691.11	65,748.34
4	56,927.60	122,890.50
5	55,231.36	127,072.20
6	100,017.30	83,156.94
7	39,627.27	62,814.67
8	142,580.20	77,149.16
9	19,771.09	55,040.31
10	4,713.12	58,223.27
11	64,098.40	62,260.37
12	44,152.12	2,958.44
13	94,835.89	106,747.10
14	144,228.00	60,995.88
15	27,172.94	26,261.61
16	87,970.65	100,813.90
17	34,361.18	55,860.60
18	119,467.30	253,713.80
19	231,571.50	126,074.50
20	70,156.00	6,766.91
21	56,651.36	320,284.40
22	179,236.60	122,682.30
